# Comparative efficacy and safety of rhTPO, romiplostim, and eltrombopag in the treatment of pediatric primary immune thrombocytopenia: a systematic review and network meta-analysis

**DOI:** 10.3389/fimmu.2025.1595774

**Published:** 2025-06-06

**Authors:** Xiaofang Zhang, Yuan Zhao, Minghang Yang, Xiaochun Feng

**Affiliations:** ^1^ College of Traditional Chinese Medicine, Changchun University of Chinese Medicine, Changchun, China; ^2^ Department of Pediatrics, Hospital Affiliated to Changchun University of Chinese Medicine, Changchun, China; ^3^ Financial Office, Affiliated Hospital of Changchun University of Chinese Medicine, Changchun, China

**Keywords:** pediatric immune thrombocytopenia, romiplostim, eltrombopag, recombinant human thrombopoietin, systematic review, network meta-analysis

## Abstract

**Background:**

Pediatric primary immune thrombocytopenia (ITP) is an autoimmune disorder characterized by isolated thrombocytopenia and an increased risk of bleeding. Conventional therapies, while effective in some cases, are often limited by suboptimal response rates and significant adverse effects with prolonged use. Thrombopoietin receptor agonists (TPO-RAs), including recombinant human thrombopoietin (rhTPO), romiplostim, and eltrombopag, have emerged as promising therapeutic alternatives for pediatric ITP. However, a comprehensive comparison of their efficacy and safety profiles remains lacking.

**Objective:**

To conduct a systematic review and network meta-analysis to evaluate and compare the efficacy and safety of rhTPO, romiplostim, and eltrombopag in the treatment of pediatric ITP.

**Methods:**

A systematic literature search was performed across PubMed, Embase, Cochrane Library, and other relevant databases. Seven randomized controlled trials (RCTs) involving a total of 375 pediatric ITP patients were included. Direct meta-analysis and Bayesian network meta-analysis were employed to assess overall response rates (ORR) and the incidence of serious adverse events (SAEs). The Surface Under the Cumulative Ranking Curve (SUCRA) was utilized to rank the interventions based on their efficacy and safety.

**Results:**

Direct meta-analysis demonstrated that romiplostim (OR = 17.57, 95% CI: 4.90–63.03), eltrombopag (OR = 5.34, 95% CI: 2.50–11.39), and rhTPO (OR = 5.32, 95% CI: 2.03–13.96) were all significantly more effective than placebo in achieving ORR (P < 0.001). In terms of SAEs, romiplostim was associated with a higher risk (OR = 3.79, 95% CI: 0.66–21.85), whereas eltrombopag (OR = 0.68, 95% CI: 0.23–2.03) and rhTPO (OR = 0.28, 95% CI: 0.01–7.17) exhibited more favorable safety profiles. Network meta-analysis ranked romiplostim (SUCRA = 0.96) as the most efficacious intervention, followed by eltrombopag (0.52) and rhTPO (0.52). For safety, rhTPO (SUCRA = 0.78) ranked highest, followed by eltrombopag (0.66), while romiplostim (0.12) was associated with the highest risk.

**Conclusion:**

Romiplostim exhibits superior efficacy in the management of pediatric ITP but necessitates vigilant monitoring for potential adverse effects, including bone marrow fibrosis. rhTPO, with its favorable safety profile, is particularly well-suited for acute bleeding scenarios. Eltrombopag offers a balanced combination of oral convenience and safety, making it an optimal choice for long-term therapy. Clinical decision-making should be guided by individual patient factors, including bleeding risk, treatment adherence, and drug accessibility. Future research should prioritize head-to-head comparative trials and long-term follow-up studies to further refine therapeutic strategies and optimize outcomes in pediatric ITP.

## 1 Introduction

Primary immune thrombocytopenia (ITP) in children is an autoimmune disorder characterized by isolated thrombocytopenia (platelet count <100×10^9^/L) and an increased propensity for bleeding (1). The pathophysiology of ITP involves antibody-mediated platelet destruction and impaired megakaryopoiesis, leading to a heterogeneous clinical spectrum ranging from asymptomatic thrombocytopenia to life-threatening intracranial hemorrhage (2). While the majority of pediatric ITP cases follow a self-limiting course, approximately 20%-30% of patients progress to persistent or chronic disease, necessitating prolonged therapeutic intervention (3). Conventional therapies, including corticosteroids, intravenous immunoglobulin (IVIg), and anti-D immunoglobulin, demonstrate rapid platelet count elevation; however, their efficacy is suboptimal, with response rates of 50%-70%, and long-term use is associated with significant adverse effects such as infections and osteoporosis (4). Splenectomy and immunosuppressive agents (e.g., rituximab) are limited by procedural risks and immunosuppression-related complications, respectively (5).

In recent years, thrombopoietin receptor agonists (TPO-RAs) have emerged as a cornerstone in the management of thrombocytopenia across various etiologies, offering effective reduction in bleeding risk, decreased reliance on platelet transfusions, and avoidance of transfusion-related adverse events (6). TPO-RAs encompass recombinant human thrombopoietin (rhTPO) and TPO-RAs, which exert their effects by binding to the thrombopoietin (TPO) receptor, thereby stimulating megakaryocyte proliferation, differentiation, and maturation to enhance platelet production (7, 8). The two most widely utilized TPO-RAs in clinical practice are romiplostim and eltrombopag (9, 10). Eltrombopag, the only oral TPO-RA approved by the U.S. Food and Drug Administration (FDA) for pediatric use (≥1 year), has demonstrated robust efficacy in multiple randomized controlled trials (11). Romiplostim, a subcutaneous peptibody, has also shown favorable response rates in children with chronic ITP (12). rhTPO, characterized by its rapid onset of action (1–2 weeks), is particularly advantageous in managing acute bleeding episodes in pediatric patients (13).

Despite their widespread use, direct comparative data on the efficacy and safety of rhTPO, romiplostim, and eltrombopag in pediatric ITP remain scarce. Existing evidence is predominantly derived from single-agent clinical trials or retrospective analyses with limited sample sizes and short follow-up durations, precluding a comprehensive assessment of long-term outcomes and risks. This gap in evidence underscores the critical need for well-designed comparative studies that can directly evaluate the relative benefits and adverse effect profiles of these agents in pediatric populations. Such analyses would not only guide optimal treatment selection but also inform individualized risk-benefit discussions, especially in cases where long-term therapy may be required. In the absence of head-to-head trials, meta-analyses and indirect comparisons offer a valuable interim approach to synthesizing existing data and identifying meaningful trends that warrant further investigation. Therefore, this study aims to conduct a systematic review and network meta-analysis to compare the efficacy and safety profiles of rhTPO, romiplostim, and eltrombopag in pediatric ITP, thereby providing evidence-based insights to inform clinical decision-making.

## 2 Methods

### 2.1 Literature search strategy

A comprehensive search was conducted across PubMed, Embase, Cochrane Library, ClinicalTrials.gov, CNKI, Wanfang Data, and VIP databases to identify prospective randomized controlled trials (RCTs) evaluating the use of eltrombopag, romiplostim, and rhTPO in pediatric ITP patients. The search timeframe spanned from the inception of each database to March 2025, with language restrictions to English and Chinese. Search terms included: (1) Disease-related: “pediatric immune thrombocytopenia,” “ITP,” “primary thrombocytopenic purpura”; (2) Interventions: “eltrombopag,” “romiplostim,” “recombinant human thrombopoietin,” “rhTPO,” “thrombopoietin receptor agonists,” “TPO-RAs.”

### 2.2 Inclusion criteria

Studies were selected based on the PICOS framework: (1) Patients (P): Children ≤18 years diagnosed with ITP according to the International ITP Working Group criteria; (2) Interventions (I): Monotherapy with rhTPO, romiplostim, or eltrombopag; (3) Controls (C): Placebo or alternative therapies (e.g., corticosteroids, immunoglobulins); (4) Outcomes (O): Response rate (platelet count ≥50×10^9^/L in the absence of severe bleeding), incidence of serious adverse events (e.g., hepatotoxicity, thromboembolic events).

Exclusion criteria included non-clinical studies (e.g., reviews, case reports), studies involving patients with concurrent hematologic disorders or significant comorbidities (e.g., malignancies, autoimmune diseases), and studies with incomplete data or insufficient detail for analysis.

### 2.3 Data extraction

Two independent investigators extracted data using a standardized template, recording study characteristics (author, publication year, study design, sample size, intervention protocol), patient demographics (age, sex, baseline platelet count, bleeding severity, prior treatment lines), and outcome measures (overall response rate [ORR], complete response [CR] rate, platelet count dynamics, bleeding events, adverse events). Discrepancies were resolved through consensus or third-party adjudication.

### 2.4 Quality assessment

The methodological quality of included RCTs was assessed using the Cochrane Risk of Bias Tool, which evaluates six domains: random sequence generation, allocation concealment, blinding, completeness of outcome data, selective reporting, and other biases. Each domain was rated as “low risk,” “unclear,” or “high risk.” Two investigators independently performed the assessments, with discrepancies resolved through discussion or third-party arbitration.

### 2.5 Statistical analysis

Direct Meta-Analysis. A direct meta-analysis was conducted to compare the efficacy and safety of rhTPO, romiplostim, and eltrombopag against placebo or alternative therapies. Dichotomous outcomes—including overall response rate (ORR, defined as the proportion of patients achieving either a complete or partial platelet response) and complete response (CR) rate (defined as achieving a sustained platelet count above a predefined threshold without bleeding)—were expressed as odds ratios (ORs) with 95% credible intervals (CrIs). Heterogeneity was assessed using the Q test (P < 0.1 indicating significance) and the I² statistic. A fixed-effects model was applied if heterogeneity was low (I² ≤ 50%), and a random-effects model was used otherwise. Publication bias was evaluated using funnel plot symmetry and Egger’s test (P < 0.05 indicating bias).

Network Meta-Analysis. To synthesize both direct and indirect evidence, a Bayesian network meta-analysis (NMA) was performed using a random-effects model in Stata. Markov Chain Monte Carlo (MCMC) methods accounted for between-study heterogeneity. The same dichotomous outcomes (ORR and CR rate) were reported as ORs with 95% CrIs. Treatment efficacy was ranked using the Surface Under the Cumulative Ranking Curve (SUCRA), where values closer to 1 indicated higher likelihood of being the most effective treatment. Probability rank plots were generated to illustrate the likelihood of each intervention being the optimal therapeutic choice.

## 3 Results

### 3.1 Literature screening process

**Figure 1:**
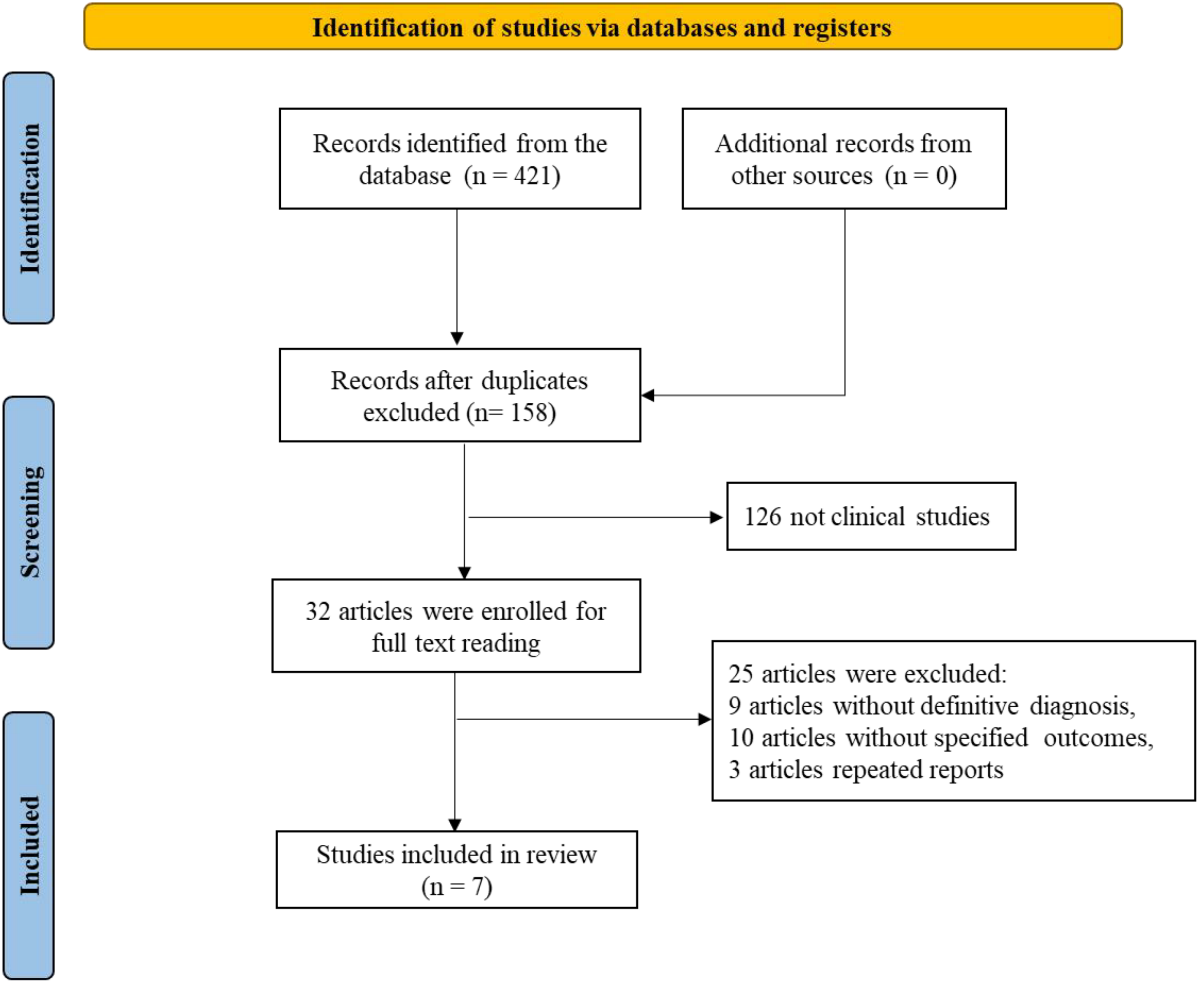
PRISMA flow diagram of literature screening.

A total of 421 records were retrieved from databases (with no additional records from other sources). After removing duplicates, 158 records remained. Subsequently, 126 non-clinical studies were excluded, leaving 32 articles for full-text review. Of these, 24 were excluded due to the absence of clear diagnostic criteria (9 articles), unspecified outcomes (10 articles), or duplicate reporting (2 articles). Ultimately, 8 studies met the inclusion criteria and were included in the analysis. The literature screening flowchart is presented in **Figure 1**.

### 3.2 Basic characteristics of included studies

This analysis included 7 clinical studies involving a total of 375 pediatric ITP patients. The interventions evaluated were Romiplostim (2 studies), Eltrombopag (3 studies), and rhTPO (2 studies). The age range of participants was 1–17 years, with female representation ranging from 27% to 56.5%. Baseline platelet counts ranged from 10.5 to 17.8 × 10^9^/L. Detailed characteristics of the included studies are summarized in **Table 1**.

**Table 1:** Basic characteristics of included studies.

First Author	Publication Year	Sample Size	Patient Age	Patient Gender	Intervention Method	Baseline Platelet Count
Bussel JB (14)	2011	22	1–17 years (median 10 years)	6 female (27%), 16 male (73%)	Romiplostim (1–10 μg/kg, weekly subcutaneous injection) vs. placebo	Median 13 × 10^9^/L
Tarantino MD (15)	2016	62	1–17 years (median 10 years)	Not specified	Romiplostim (1–10 μg/kg, weekly subcutaneous injection) vs. placebo	Median 17.8 × 10^9^/L
Elalfy MS (16)	2011	18	2.5–6 years (median 8.5 years)	5 female (28%), 13 male (72%)	Romiplostim (1–5 μg/kg, weekly subcutaneous injection) vs. placebo	Median 10.5 × 10^9^/L
Ma J (17)	2024	56	6–17 years (mean 9.8 years)	30 male (53.6%), 26 female (46.4%)	rhTPO (300 U/kg/day, subcutaneous injection for 14 days) vs. placebo	Mean 17.7 × 10^9^/L
Grainger JD (18)	2015	92	1–17 years (mean 9.4 years)	Not specified	Eltrombopag (oral, dose-adjusted to target 50–200 × 10^9^/L) vs. placebo	Mean 17.8 × 10^9^/L
Bussel JB (19)	2015	67	1–17 years (median 13 years)	Not specified	Eltrombopag (oral, dose-adjusted to target 50–200 × 10^9^/L) vs. placebo	Mean 15.5 × 10^9^/L
Lu YY (20)	2018	58	1–12 years (mean 6–7 years)	26 male (44.8%), 32 female (55.2%)	rhTPO group: rhTPO (300 U/kg/day, subcutaneous injection for 14 days) + dexamethasone (0.6 mg/kg/day, intravenous infusion for 4 days, repeated every 28 days); DXM group: dexamethasone (same as intervention group)	Mean 12 × 10^9^/L

**Figure 2:**
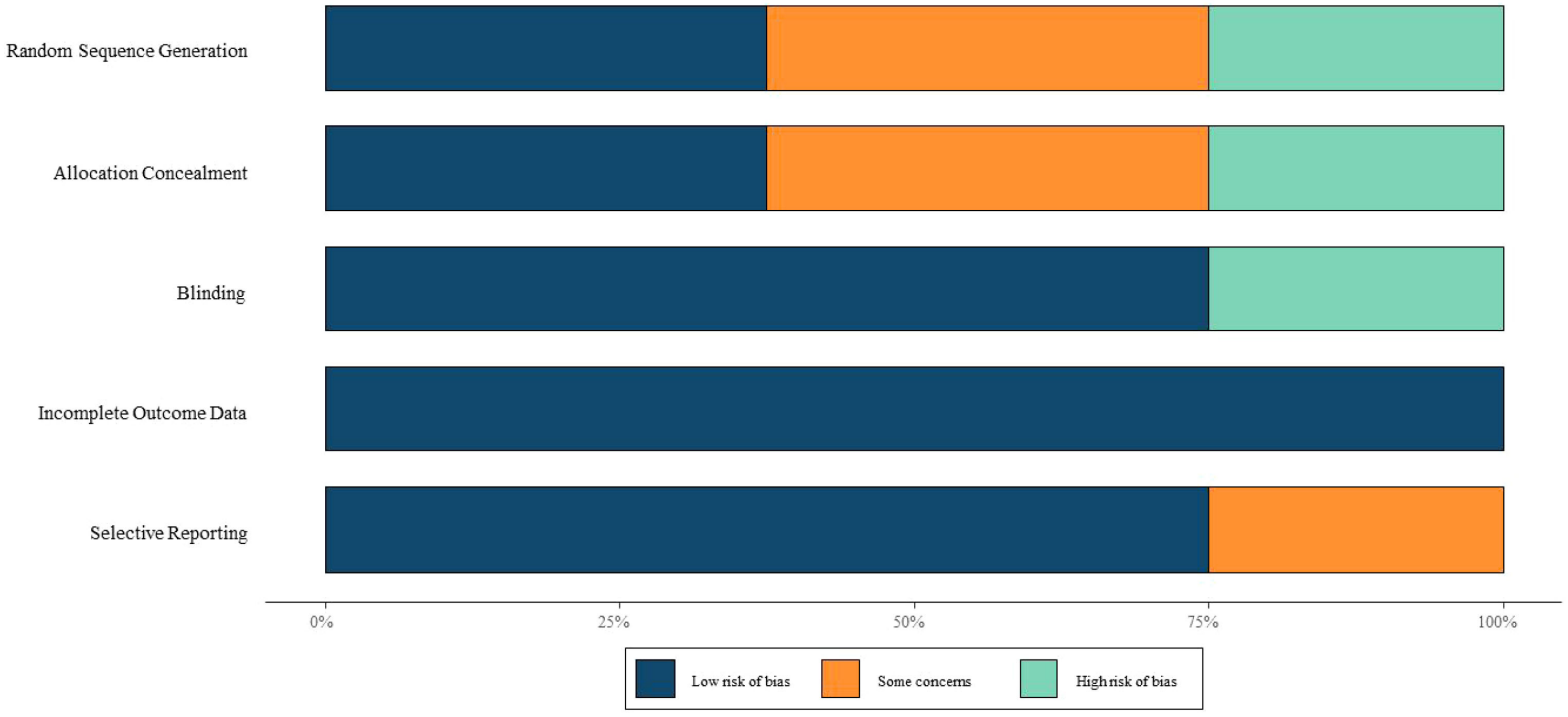
Risk of bias.

Regarding study quality, Tarantino MD (2016), Grainger JD (2015), and Bussel JB (2015) were assessed as high-quality studies. In contrast, Elalfy MS (2011) and Lu YY (2018) were deemed to have a high risk of bias due to unclear randomization methods and lack of blinding. Most studies exhibited uncertainties in random sequence generation, allocation concealment, and blinding, necessitating cautious interpretation of the results. The risk of bias assessment is illustrated in **Figure 2**.

### 3.3 Direct meta-analysis results

#### 3.3.1 ORR

**Figure 3:**
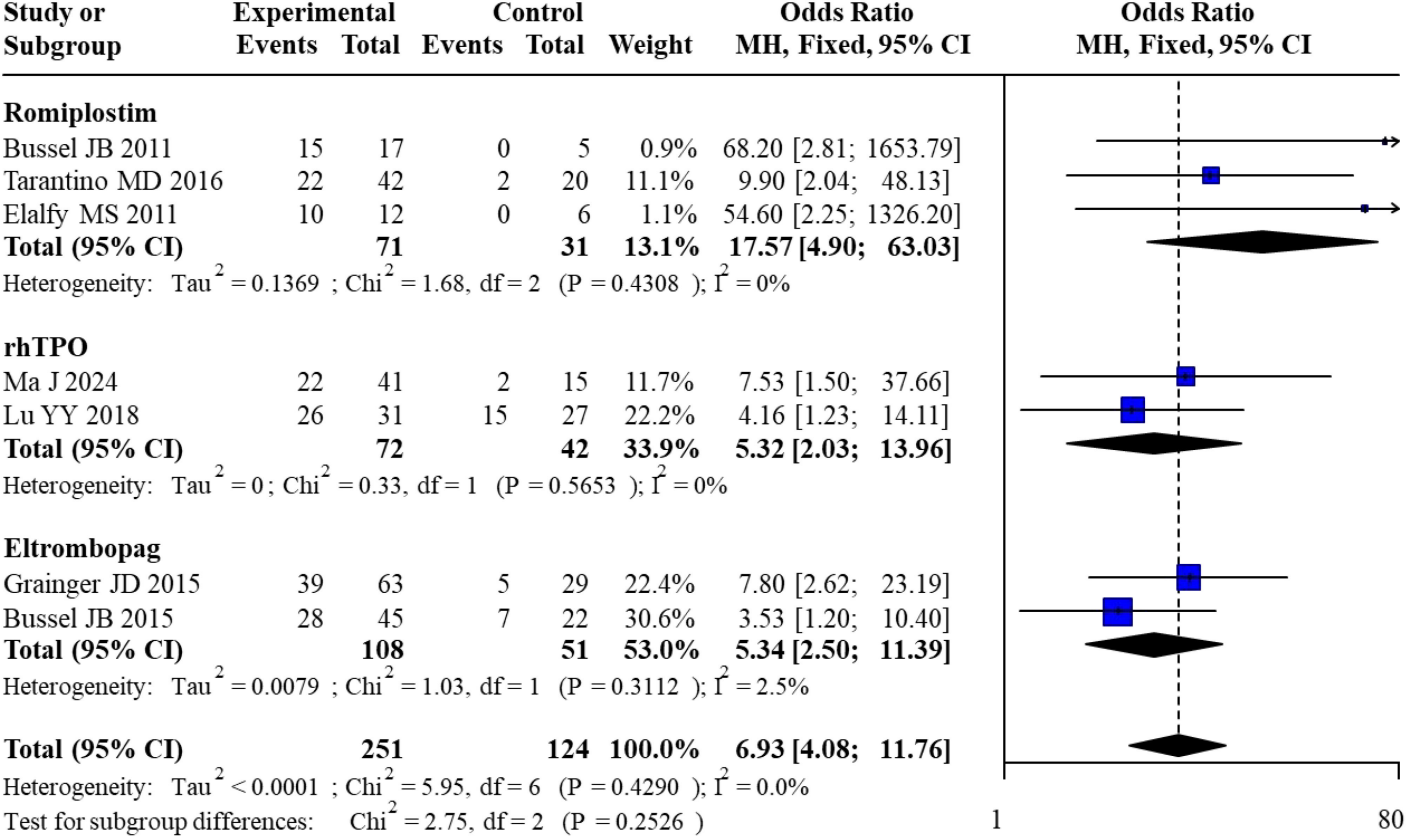
Forest plot of direct meta-analysis for overall response rate.

All 7 included studies reported overall response rates. For Romiplostim, pooled analysis of 3 studies yielded an OR of 17.57 (95% CI: 4.90–63.03) with no heterogeneity (I² = 0%). For rhTPO, pooled analysis of 2 studies resulted in an OR of 5.32 (95% CI: 2.03–13.96), also with no heterogeneity (I² = 0%). For Eltrombopag, pooled analysis of 2 studies showed an OR of 5.34 (95% CI: 2.50–11.39) with minimal heterogeneity (I² = 2.5%). The overall pooled OR across all studies was 6.93 (95% CI: 4.08–11.76) with no heterogeneity (I² = 0%). Subgroup analysis revealed no statistically significant differences between subgroups (χ² = 2.75, df = 2, P = 0.2526). The forest plot for the direct meta-analysis of overall response rates is presented in **Figure 3**.

**Figure 4:**
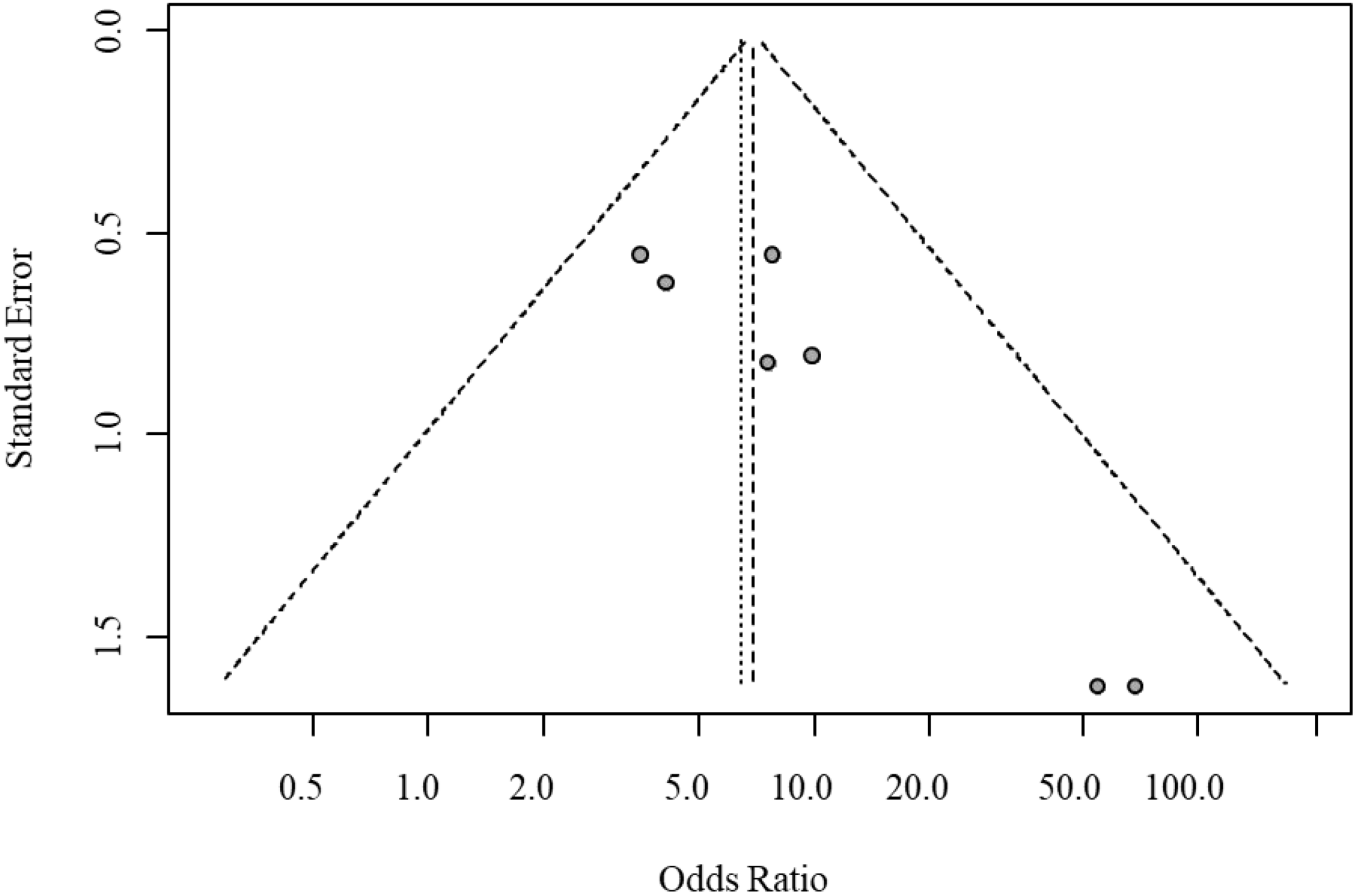
Funnel plot for publication bias of overall response rate.

Egger’s test indicated significant funnel plot asymmetry (t = 4.10, P = 0.0094), suggesting potential small-study bias. The bias estimate was 2.33 (SE = 0.569), indicating that smaller studies tended to report larger treatment effects. Heterogeneity testing revealed moderate between-study heterogeneity (tau² = 0.273); however, sensitivity analysis excluding high-heterogeneity studies confirmed the robustness of the results. The funnel plot for publication bias in overall response rates is shown in **Figure 4**.

#### 3.3.2 Serious adverse events

**Figure 5:**
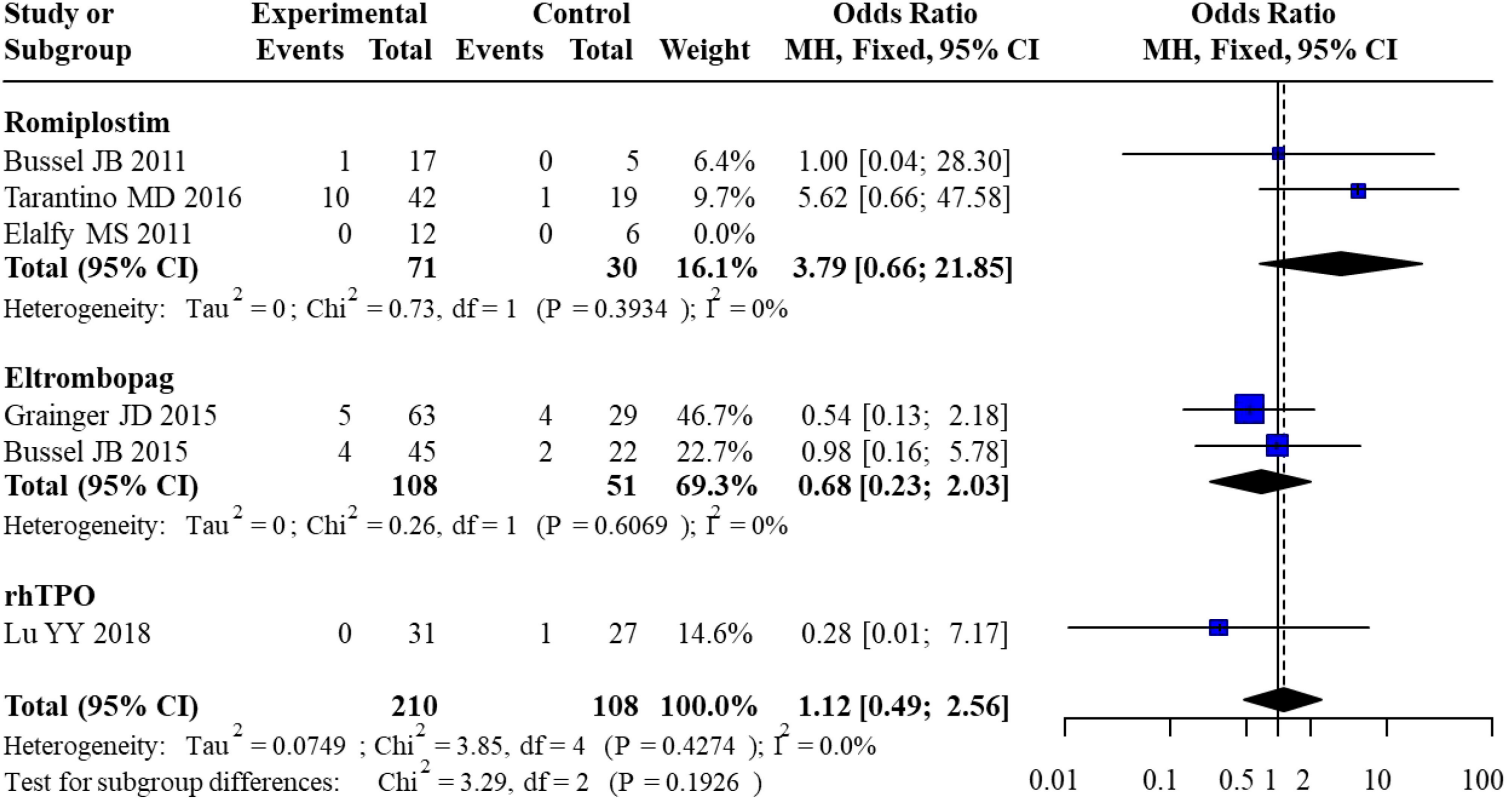
Forest plot of direct meta-analysis for serious adverse events.

Six studies reported the incidence of serious adverse events. For Romiplostim, pooled analysis of 3 studies yielded an OR of 3.79 (95% CI: 0.66–21.85) with no heterogeneity (I² = 0%). For Eltrombopag, pooled analysis of 2 studies resulted in an OR of 0.68 (95% CI: 0.23–2.03), also with no heterogeneity (I² = 0%). For rhTPO, only 1 study was included, with an OR of 0.28 (95% CI: 0.01–7.17). The overall pooled OR across all studies was 1.12 (95% CI: 0.49–2.56) with no heterogeneity (I² = 0%). Subgroup analysis revealed no statistically significant differences in SAE risk between subgroups (χ² = 3.29, df = 2, P = 0.1926). The forest plot for the direct meta-analysis of serious adverse events is presented in **Figure 5**.

**Figure 6:**
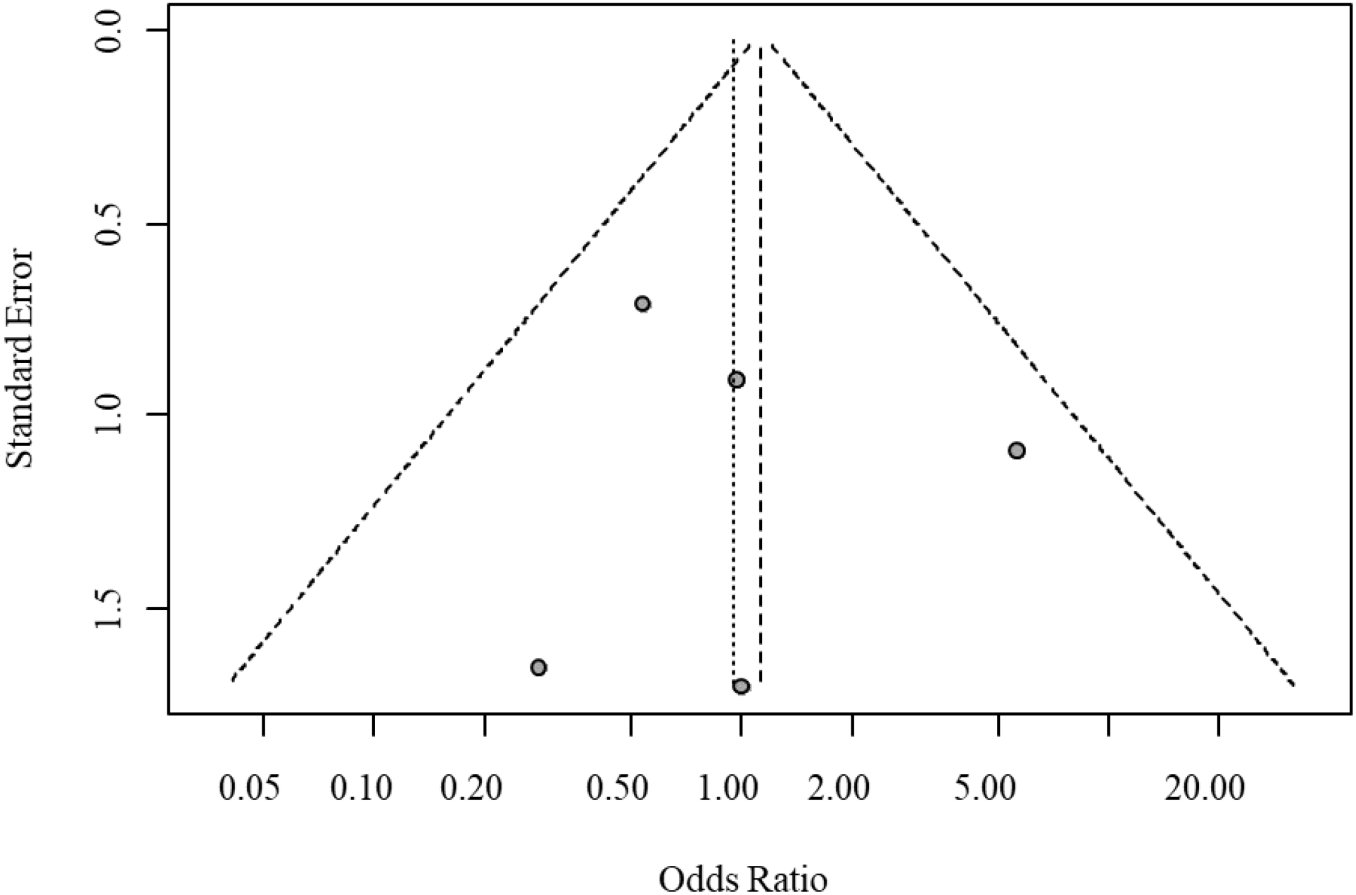
Funnel plot for publication bias of serious adverse events.

Egger’s test indicated no significant funnel plot asymmetry (t = 0.22, P = 0.8422), with a bias estimate of 0.3430 (SE = 1.5813). Visual inspection of the funnel plot further confirmed the absence of significant publication bias or small-study bias. The funnel plot for publication bias in serious adverse events is shown in **Figure 6**.

### 3.4 Network meta-analysis

#### 3.4.1 Network geometry

**Figure 7:**
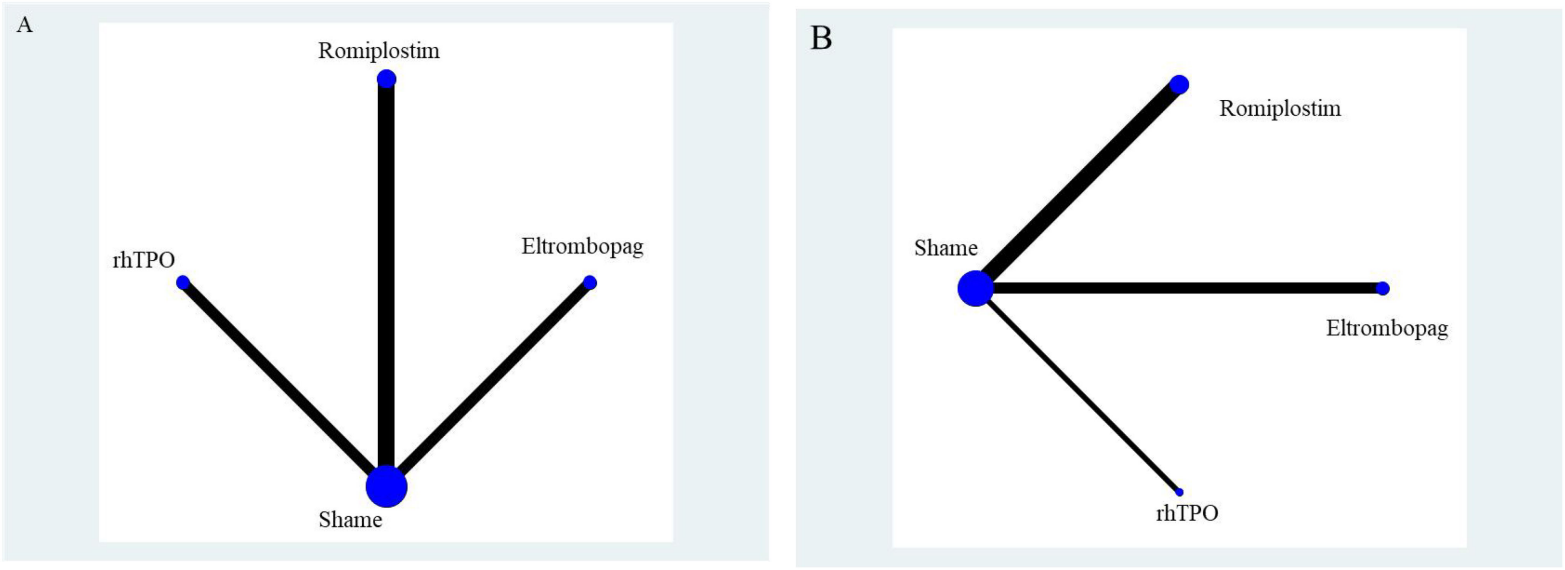
Network geometry diagram. **(A)** represents overall response rate, and **(B)** represents serious adverse events.

The network geometry diagram illustrates the interventions as nodes and direct comparisons as connecting lines. For both overall response rates and serious adverse events, direct comparisons were available between Romiplostim, Eltrombopag, rhTPO, and placebo (Sham). However, no direct comparisons were available between the active interventions, necessitating indirect comparisons to evaluate their relative efficacy and safety. The network geometry diagram is presented in **Figure 7**.

#### 3.4.2 SUCRA rankings for overall response rate and serious adverse events

All 7 studies reported overall response rates, including 3 studies comparing Romiplostim with placebo, 2 studies comparing Eltrombopag with placebo, and 2 studies comparing rhTPO with placebo. Based on SUCRA rankings, Romiplostim (SUCRA = 0.96) had the highest probability of being the most effective intervention, followed by rhTPO (SUCRA = 0.52) and Eltrombopag (SUCRA = 0.52). Placebo (Sham) ranked last (SUCRA = 0.00).

**Figure 8:**
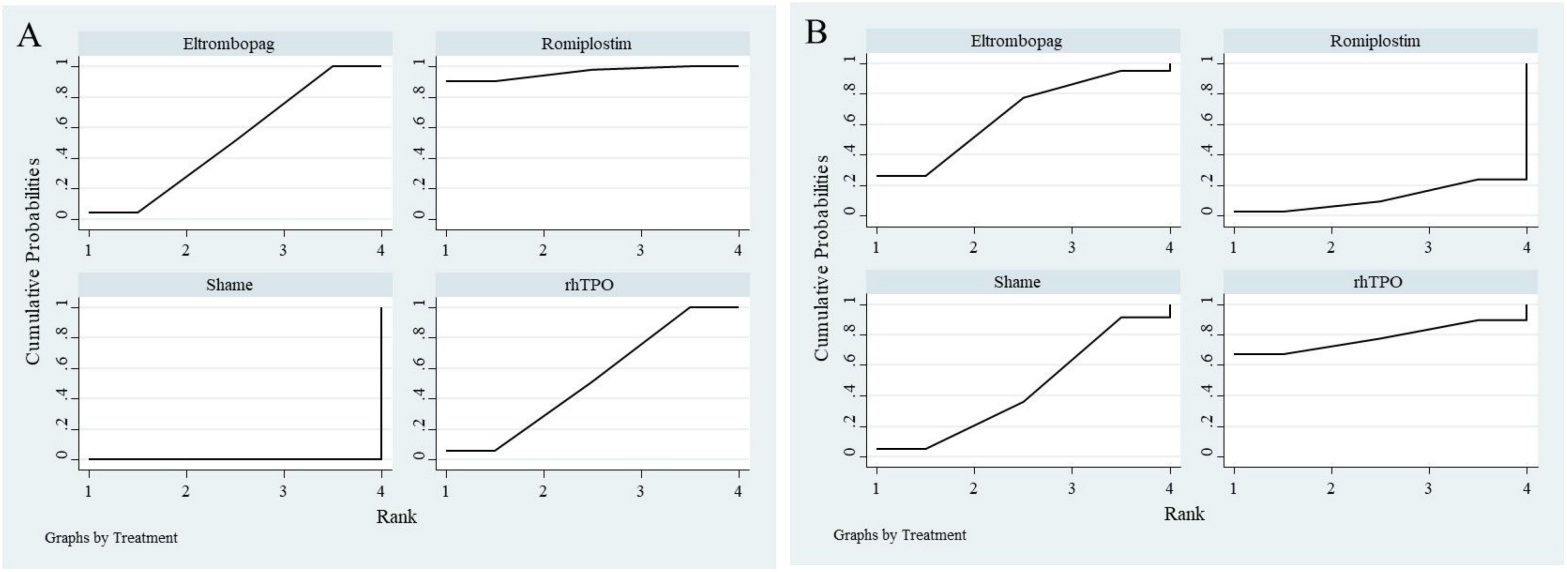
SUCRA ranking plot from network meta-analysis. **(A)** represents overall response rate, and **(B)** represents serious adverse events.

For serious adverse events, 6 studies were included, with 3 comparing Romiplostim with placebo, 2 comparing Eltrombopag with placebo, and 1 comparing rhTPO with placebo. Based on SUCRA rankings, rhTPO (SUCRA = 0.78) had the highest probability of being the safest intervention, followed by Eltrombopag (SUCRA = 0.66) and placebo (Sham) (SUCRA = 0.44). Romiplostim ranked last (SUCRA = 0.12). The SUCRA rankings for network meta-analysis are illustrated in **Figure 8**.

## 4 Discussion

The therapeutic paradigm for pediatric primary ITP has evolved significantly with the introduction of TPO-RAs. This study represents the first systematic review and network meta-analysis to comprehensively evaluate the comparative efficacy and safety of three TPO-RAs—romiplostim, rhTPO, and eltrombopag—in pediatric ITP. The findings reveal that romiplostim demonstrates superior efficacy, as evidenced by its high overall response rate (OR = 17.57) and SUCRA value (0.96), while rhTPO exhibits the most favorable safety profile (SUCRA = 0.78). Eltrombopag, in contrast, offers a balanced profile between efficacy and safety, providing clinicians with critical insights for individualized therapeutic decision-making in pediatric ITP.

Romiplostim, a long-acting peptide-based TPO-RA, achieves its therapeutic effects through sustained stimulation of megakaryocyte proliferation via subcutaneous administration. Its efficacy advantage is likely attributable to its unique pharmacokinetic properties, including a prolonged half-life of approximately 50 hours, which significantly exceeds that of eltrombopag (21–35 hours) and rhTPO (3–4 hours) (21, 22). This extended half-life may enhance its ability to maintain stable platelet counts over time. Although the forest plot from the meta-analysis suggests an elevated odds ratio for adverse events associated with romiplostim (OR = 3.79), the confidence interval crosses the line of null effect, indicating that this finding is not statistically significant. Despite this, the observed trend, coupled with the biological rationale linking prolonged megakaryocyte stimulation to the development of bone marrow fibrosis, justifies the clinical concern. This aligns with findings from Li et al. (23), who reported fibrotic complications in the context of romiplostim use. Therefore, while the data do not yet definitively confirm an increased risk, they underscore the need for ongoing monitoring and further high-quality studies to better delineate the long-term safety profile of romiplostim, particularly in relation to marrow pathology.

In contrast, rhTPO, a recombinant form of endogenous thrombopoietin, is characterized by rapid onset of action and efficient metabolic clearance, potentially mitigating toxicity related to drug accumulation (24). Eltrombopag, the only oral TPO-RA, demonstrates comparable efficacy to rhTPO and a favorable safety profile, with no significant increase in the risk of hepatotoxicity (OR = 0.68) compared to placebo. Its oral bioavailability and flexible dosing regimen make it particularly suitable for long-term management, especially in younger children or those with needle aversion (25).

These findings are consistent with the American Society of Hematology (ASH) guidelines, which endorse TPO-RAs as the preferred second-line therapy due to their superior response rates (60–80%) compared to alternatives such as rituximab (20–30%) (26). However, the European LeukemiaNet guidelines emphasize the importance of cost-effectiveness, noting that eltrombopag is approximately 40% less expensive than romiplostim, a consideration that is particularly relevant in resource-limited settings. Additionally, Chinese expert consensus underscores the utility of rhTPO in acute settings due to its rapid onset of action (1–2 weeks), making it a preferred choice for managing acute bleeding episodes (27, 28).

The observed differences in efficacy among these agents can be attributed to their distinct mechanisms of action. Romiplostim, an Fc-peptide fusion TPO-RA, binds to the transmembrane domain of the TPO receptor, activating the JAK2/STAT5 signaling pathway to promote megakaryocyte proliferation (21). Its extended half-life is facilitated by Fc-mediated recycling, while subcutaneous administration ensures consistent plasma concentrations (29). Eltrombopag, a small-molecule non-peptide TPO-RA, competitively binds to the extracellular domain of the TPO receptor, activating downstream signaling pathways. However, its oral administration may result in variable plasma concentrations, potentially impacting efficacy stability (30). rhTPO, as a recombinant analog of endogenous thrombopoietin, directly mimics physiological TPO activity, but its short half-life necessitates frequent dosing, which may compromise adherence in chronic ITP management (31).

From a clinical perspective, romiplostim is particularly suited for children with chronic ITP who require rapid and sustained platelet count elevation, especially those refractory to corticosteroids or IVIg. However, its use necessitates vigilant monitoring for bone marrow fibrosis, with bone marrow biopsies recommended at 6- to 12-month intervals. Eltrombopag, owing to its oral administration, is an ideal option for long-term, home-based therapy, particularly in younger children or those with needle phobia (25, 32). rhTPO is preferred in acute settings, such as severe bleeding or perioperative scenarios requiring rapid platelet elevation, though its higher cost (approximately 1.5 times that of eltrombopag) may limit its accessibility in resource-constrained environments (33).

This study is not without limitations. First, the reliance on indirect comparisons, particularly the absence of head-to-head trials comparing romiplostim and eltrombopag, limits the strength of the evidence. Second, the relatively short follow-up duration (median 24 weeks) precludes the assessment of long-term safety outcomes, such as carcinogenicity. Third, the observed heterogeneity may stem from variations in dosing regimens (e.g., romiplostim 2–10 μg/kg) and patient populations, which could influence the results. Future research should prioritize: (1) Conducting multicenter, head-to-head randomized controlled trials comparing eltrombopag and romiplostim; (2) Investigating predictive biomarkers (e.g., TPO receptor gene polymorphisms) to enable precision medicine approaches; (3) Establishing real-world cohorts to evaluate the pharmacoeconomic impact of these therapies.

## 5 Conclusion

This study establishes that romiplostim offers the highest efficacy in the treatment of pediatric ITP, though its potential safety risks, particularly the association with bone marrow fibrosis, warrant careful consideration. rhTPO and eltrombopag play pivotal roles in acute-phase management and long-term therapy, respectively. Clinical decision-making should integrate factors such as bleeding risk, treatment adherence, and drug accessibility, while also incorporating individualized biomarkers and long-term safety data. Future research should focus on high-quality head-to-head trials and real-world studies to further refine and optimize therapeutic strategies for pediatric ITP.

## Data Availability

The original contributions presented in the study are included in the article/supplementary material. Further inquiries can be directed to the corresponding author.
